# Loss of Function of the Gene Encoding the Histone Methyltransferase KMT2D Leads to Deregulation of Mitochondrial Respiration

**DOI:** 10.3390/cells9071685

**Published:** 2020-07-13

**Authors:** Consiglia Pacelli, Iolanda Adipietro, Natascia Malerba, Gabriella Maria Squeo, Claudia Piccoli, Angela Amoresano, Gabriella Pinto, Pietro Pucci, Ji-Eun Lee, Kai Ge, Nazzareno Capitanio, Giuseppe Merla

**Affiliations:** 1 Department of Clinical and Experimental Medicine, University of Foggia, 71121 Foggia, Italy; consiglia.pacelli@unifg.it (C.P.); claudia.piccoli@unifg.it (C.P.); 2 Division of Medical Genetics, Fondazione IRCCS Casa Sollievo della Sofferenza, 71013 San Giovanni Rotondo (FG), Italy; iole.adipietro@gmail.com (I.A.); n.malerba@operapadrepio.it (N.M.); g.squeo@operapadrepio.it (G.M.S.); 3 Department of Chemical Sciences, University of Naples “Federico II”, 80126 Napoli, Italy; angela.amoresano@unina.it (A.A.); gabriella.pinto@unina.it (G.P.); pucci@unina.it (P.P.); 4 INBB—Biostructures and Biosystems National Institute, 00136 Roma, Italy; 5 Department of Chemical Sciences, CEINGE Advanced Biotechnology, Federico II University, 80145 Napoli, Italy; 6 National Institute of Diabetes and Digestive and Kidney Diseases, National Institutes of Health, Bethesda, MD 20892, USA; leejieun@niddk.nih.gov (J.E.L.); kaig@niddk.nih.gov (K.G.)

**Keywords:** Kabuki syndrome, *KMT2D*, mitochondria

## Abstract

*KMT2D* encodes a methyltransferase responsible for histone 3 lysine 4 (H3K4) mono-/di-methylation, an epigenetic mark correlated with active transcription. Here, we tested the hypothesis that *KMT2D* pathogenic loss-of-function variants, which causes the Kabuki syndrome type 1, could affect the mitochondrial metabolic profile. By using Seahorse technology, we showed a significant reduction of the mitochondrial oxygen consumption rate as well as a reduction of the glycolytic flux in both *Kmt2d* knockout MEFs and skin fibroblasts of Kabuki patients harboring heterozygous *KMT2D* pathogenic variants. Mass-spectrometry analysis of intermediate metabolites confirmed alterations in the glycolytic and TCA cycle pathways. The observed metabolic phenotype was accompanied by a significant increase in the production of reactive oxygen species. Measurements of the specific activities of the mitochondrial respiratory chain complexes revealed significant inhibition of CI (NADH dehydrogenase) and CIV (cytochrome c oxidase); this result was further supported by a decrease in the protein content of both complexes. Finally, we unveiled an impaired oxidation of glucose and larger reliance on long-chain fatty acids oxidation. Altogether, our findings clearly indicate a rewiring of the mitochondrial metabolic phenotype in the KMT2D-null or loss-of-function context that might contribute to the development of Kabuki disease, and represents metabolic reprogramming as a potential new therapeutic approach.

## 1. Introduction

Growing evidence highlights the tight interaction between metabolism and epigenetics [1]. Covalent modification of DNA and histones require, indeed, the availability of amino acid derivatives, cofactors, and central metabolites to feed the plethora of epigenetic-modifying enzymes. Other ways to induce epigenetic modulations, such as chromatin remodeling, also impact the metabolic phenotype. However, the mechanisms that confer selectivity of chromatin remodeling in regulating the metabolic phenotype remain to be fully understood.

Histone-lysine N-methyltransferase 2D (KMT2D) belongs to the SET1 family of histone H3K4 methyltransferases and is part of the ASCOM multi-protein complex that catalyzes mono-, di-, and tri-methylation of H3K4 using S-adenosyl methionine as a co-substrate [2]. KMT2D is a positive key regulator of gene expression in the context of cellular differentiation in different tissues. Although KMT2D activity can be replaced by other histone methyltransferases, its genetic knockout reduced global H3K4me1 levels, and the majority of KMT2D binding sites are localized in putative enhancer elements [3,4,5].

Pathogenic loss-of-function (lof) variants in the *KMT2D* gene with an autosomal dominant pattern of inheritance have been linked to the great majority of cases of Kabuki syndrome, which is one of the most well-characterized pediatric chromatinopathies [6] and is hallmarked by multiple congenital anomalies and intellectual disabilities [7,8]. A lower percentage of cases of Kabuki syndrome are related to pathogenic variants in the *KDM6A* gene, which encodes another important component of the ASCOM complex that is responsible for H3K27 demethylation of repressive polycomb-derived methylation marks [9]. Studies in mouse models have demonstrated that *Kmt2d* is essential for adipogenesis, myogenesis, macrophage activation, and lymphomagenesis, demonstrating additional roles for KMT2D as a mono- and di-methyltransferase at enhancers [5,10,11]. Collectively, these findings indicate that KMT2D regulates key gene expression programs via H3K4 mono-, di-, and tri-methylation, and its role depends on the cellular context and developmental stage. Intriguingly, heterozygous *Kmt2d*^+/−^ mice exhibit metabolic alterations consisting of enhanced glucose tolerance, insulin sensitivity, and increased serum bile acid [12] as well as resistance to over-nutrition-induced hepatic steatosis [13]. These lines of evidences suggest a regulatory function of KMT2D-mediated epigenetic modification on metabolism. Since mitochondria have emerged as an important source of metabolites utilized as co-substrates by both epigenetic writers and erasers [14], we investigated this issue by focusing on the role of *KMT2D* in mitochondrial aerobic metabolism. For this purpose, we used *Kmt2d* knockout MEFs and fibroblasts from Kabuki syndrome-affected patients harboring *KMT2D* heterozygous pathogenic variants. By combining biochemical and metabolomic approaches in mouse and human cell models, we demonstrated a rewiring of the mitochondrial metabolic phenotype due to KMT2D alteration that might contribute to the onset of Kabuki syndrome. 

## 2. Materials and Methods

### 2.1. Cell Samples

Primary dermal fibroblasts were obtained from skin biopsies of Kabuki patients and healthy individuals after signing the appropriate informed consent, provided by Genomic and Genetic Disorders Biobank, member of the Telethon Network of Genetic Biobanks [15]. The generation and use of skin fibroblasts were reviewed and approved by Ethical Committee at Fondazione IRCCS Casa Sollievo della Sofferenza (14/11/2018, 156/CE). Cells were grown in minimum essential medium supplemented with 1% glutamine, 10% FBS, and 1% PenStrep. Healthy control skin fibroblast was transiently transfected with 2 μg of *MLL2* CRISPR/Cas9 KO Plasmids (Santa Cruz Biotechnology, Dallas, TX, USA). After 48 h, GFP-positive transfected cells were enriched by FACS analysis and isolated as a single cell in a 96 multi-well plate. After approximately 20 days, single-well adherent cells were detached by trypsinization, and expanded as clonal cells. Each clone was analyzed for *KMT2D* variants by Sanger sequencing. The presence of off-targets predicted by Cas-OFFinder (http://www.rgenome.net/cas-offinder/) using default parameters [16] was ruled out by PCR and Sanger sequencing (Table S1).

Primary mouse embryonic fibroblasts (MEFs) isolated from E13.5 *Kmt2d*^f/f^ embryos [5] were immortalized by following the 3T3 protocol. Immortalized *Kmt2d*^f/f^ MEFs were infected with retroviruses expressing Cre to generate *Kmt2d*-KO MEFs. Cells were cultured in DMEM supplemented with 10% FBS and 1% PenStrep.

### 2.2. Laser Scanning Confocal Microscopy Live Imaging

Cells cultured at a low density on fibronectin-coated 35-mm glass-bottom dishes (Ibidi) were incubated for 20 min at 37 °C with 0.2 μM tetramethylrhodamine ethyl ester (TMRE, Invitrogen, Molecular probesTM, Carlsbad, CA, USA) to detect the mitochondrial membrane potential, or with 10 μM 2,7-dichlorofluorescin diacetate (DCF-DA, Invitrogen, Molecular probesTM, Carlsbad, CA, USA ) to detect cellular peroxide, or with 5 μM MitoSOX™ (Invitrogen, Molecular probesTM, Carlsbad, CA, USA) to detect mitochondrial superoxide anion. Stained cells were washed with PBS and examined by a Leica TCS SP8 confocal laser scanning microscopy system (images collected using a 60X objective [1.4 NA]). Acquisition, storage, and analysis of data were performed with LasX software from Leica or ImageJ1.48 (Wayne Rasband, NIH, USA, http://imagej.nih.gov/ij).

### 2.3. Immunoblotting

Immunoblots were performed by standard protocol; PVDF membranes were probed with MitoProfile Total OXPHOS Human WB Antibody cocktail (1:500; Abcam Cambridge, UK) and β-ACTIN (1:5000; SIGMA Aldrich, St. Louis, MO, USA). The secondary antibody was horseradish peroxidase-conjugated (1:2500; Cell Signaling Technology). The signals were developed by an enhanced chemiluminescence kit (ClarityTM Western ECL Substrate, Bio-Rad), acquired by a ChemiDoc Imaging System XRS + (BioRad), and then analyzed for densitometry with the ImageJ Lab 4.1 software.

### 2.4. RNA Isolation and Quantitative RT-PCR (RT-qPCR)

Total RNA was extracted using the RNeasy Mini Kit (Qiagen, Germany). The QuantiTect Reverse Transcription Kit (Qiagen) was used to carry out cDNA synthesis. cDNA was amplified using Power SYBR Green PCR Master Mix (Applied biosystem) on an ABI PRISM 7009HT System. The relative amounts of target genes were normalized to *GAPDH* or *RN18S* and the 2^−ΔΔCt^ method was used for calculations. Validated primers for RT-qPCR are provided upon request.

### 2.5. Metabolites Extraction

A liquid-liquid extraction was carried out for the recovery of metabolites and protein precipitation from whole lysates of wild-type (+/+), heterozygous mutant (+/−), and homozygous mutant (−/−) human cells. In detail, a solution of cold acetonitrile/methanol (50/50) containing 0.1% acetic acid was used to dilute the cellular lysates by 5-fold. The suspension was sonicated for 10 min, cooled at −20 °C, and centrifuged at 12,000 rpm for 10 min at 4 °C. The supernatant was filtered by 0.22-µm centrifugal filters (PVDF, Millipore) and analyzed by a LC-MS/MS mass spectrometer. Each sample was analyzed in duplicate. Three biological replicates were analyzed for heterozygous and homozygous mutants, and two biological replicates were analyzed for wild-type ones.

### 2.6. LC–MS/MS Analysis 

Metabolite extracts were separated by liquid chromatography using an LC Eksigent combined to a 4000 QTRAP® mass spectrometer (AB Sciex). The auto-sampler was cooled at 4 °C, the injection volume was 5 μL, and the flow rate was set to 30 μL/min. The chromatographic separation was obtained on a column Halo C18 2.7 um 90A 1x50 mm at a temperature of 38 °C. Eluent A was H_2_O and 0.1% acetic acid, whereas eluent B was a solution of 50% ACN, 50% isopropanol, and 0.1% acid acetic. Gradient conditions followed an increase of organic eluent from 0% to 90% in a 6-min run. The mass spectrometry analysis was performed in multiple reaction monitoring (MRM) mode, with an ionization source ESI. Metabolites were tuned for negative ionization polarity by using optimized values of precursor (Q1) and daughter ions (Q2) as well as collision energy (CE) and declustering potential (DP) as reported in the literature [17]. 

### 2.7. Measurements of Metabolic Fluxes

Cellular respiration (oxygen consumption rate (OCR) and extracellular acidification rate (ECAR)) were assessed using an XF96 Extracellular Flux Analyzer (Seahorse Bioscience, Billerica, MA, USA). First, 15 × 10^3^/well MEFs and 10 × 10^3^/well fibroblasts were incubated for 45 min in 180 µL of bicarbonate-free DMEM supplemented with 10 mM glucose 2 mM l-glutamine and 1 mM sodium pyruvate pre-warmed at 37 °C. The XF Cell Mito Stress Test (Seahorse Bioscience) was used to measure the key parameters of mitochondrial respiration when using specific mitochondrial inhibitors and uncouplers. Oligomycin (1μM), carbonilcyanide *p*-triflouromethoxyphenylhydrazone (FCCP) (0.5 μM), and a mixture of rotenone/antimycin A (both 1 μM) were injected sequentially according to the manufacturer’s instructions. Before drug addition, basal OCR was measured. Oligomycin was injected to inhibit ATP synthase (complex V), and OCR was recorded. To determine the maximal respiration, the uncoupler FCCP was injected. Finally, a mixture of rotenone/antimycin A was injected to inhibit the flux of electrons through complexes I and III and to enable the calculation of the spare respiratory capacity. For ECAR analysis, glycolytic flux (basal glycolysis, glycolytic capacity, and glycolytic reserve) was analyzed by the sequential addition of 10 mM glucose, 1 μM oligomycin, and 100 mM 2-deoxyglucose. The Seahorse XF Mito Fuel Flex Test Kit was used for measuring the basal state mitochondrial fuel oxidation in live cells. This assay kit utilizes a set of inhibitors to reveal the cells’ ability to switch oxidative pathways in meeting basal energetic demands and provides information regarding the relative contributions of glucose, glutamine, and long-chain fatty acid oxidation to basal respiration. The assay uses specific inhibitors in different combinations to measure the dependency, capacity, and flexibility of cells to oxidize 3 major mitochondrial fuels: Glucose (pyruvate), glutamine (glutamate), and long-chain fatty acids in the basal energetic state. The inhibitors used were: BPTES (3 μM), an inhibitor of glutaminase; etomoxir (4 μM), an inhibitor of long-chain fatty acid β-oxidation (FAO); and UK5099 (2 μM), an inhibitor of the mitochondrial pyruvate carrier. All measurements were normalized to the total protein concentration using the Pierce BCA Protein Assay Kit (Thermo Fisher Scientific).

### 2.8. Measurement of the Steady-State Content and Rates of Production of ATP

Cellular steady-state ATP content was determined using the PerkinElmer ATPlite kit (PerkinElmer) according to the manufacturer’s instructions on MEFs collected by trypsinization, pelleted at 500× *g*, and resuspended in phosphate-saline buffer. To distinguish the relative contributions to the ATP production of glycolysis, cultured cells were treated with 1.0 µM oligomycin for 10–30 min and the residual ATP level attributed to glycolysis; the difference between the ATP content measured in the absence and in the presence of oligomycin was attributed to the mitochondrial ATP-synthase. Measurements were performed with a TD 20–20 Luminometer (Turner Designs) and normalized to the protein content.

The cellular ATP production rates were quantified using the Agilent Seahorse XF ATP real-time assay kit using label-free technology in real time as specified by the manufacturer (see also [18]. Briefly, according to the equation: glucose + 2 ADP +2 Pi → 2 lactate + 2 ATP + 2 H_2_O + 2 H^+^, the glycolytic ATP production rate (glycoATP PR) was estimated from the glycolytic proton efflux rate (glycoATP PER) following determination of the buffering power (BP). BP was calculated separately by measuring pH changes elicited by consecutive additions of titrated HCl in 5 mM Hepes-supplemented DMEM, in the presence of the same number of cells assayed and was 1.79 (mmol H^+^/L/pH). The mitochondrial ATP production rate (mitoATP PR) was equal to OCR_ATP_ x 2 x P/O (mol ATP/mol O), where OCR_ATP_ was the difference between the basal OCR and the OCR in the presence of oligomycin; a theoretical P/O ratio of 2.25 was assumed based on the combined oxidation of glucose, fatty acid, and glutamine.

### 2.9. Mitochondrial Respiratory Complex Enzymatic Activity

Measurement of the specific activity of mitochondrial NADH:ubiquinone oxidoreductase (C I), cytochrome c oxidase (C IV), and citrate synthase (CS) was carried out spectrophotometrically on frozen–thawed and ultrasound-treated cells as previously described [19,20].

### 2.10. Statistical Analysis

Data are shown as mean ± SEM. Data were compared by an unpaired student’s t-test. Differences were considered statistically significant when the *p*-value was less than 0.05. All analyses were performed using Graph Pad Prism (Graph Pad software, v 6.01, San Diego, CA, USA).

## 3. Results

### 3.1. Loss of Kmt2d Causes Deregulation of the Respiratory and Glycolytic Metabolic Fluxes

**Figure 1:**
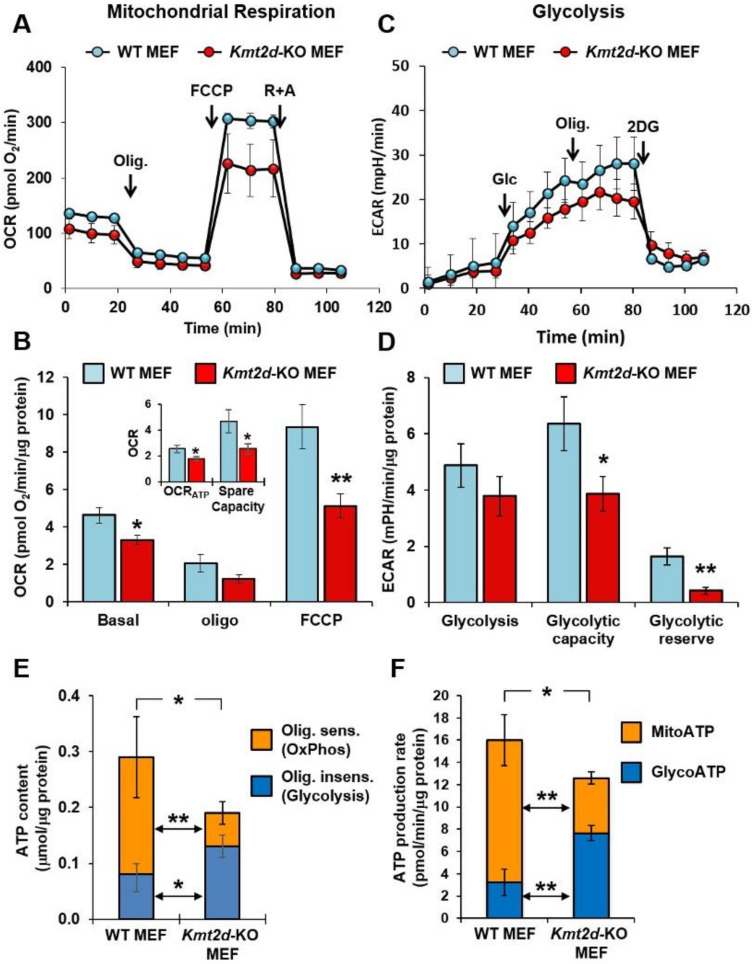
Metabolic fluxes analysis of WT and Kmt2d-KO MEF. Cells were analyzed in microplates by Seahorse XP 96 according to the Mito Stress protocol of the manufacturer. (**A**) and (**C**) are representative traces of the oxygen consumption rates (OCRs) and extracellular acidification rates (ECARs), respectively. Where indicated, the following compounds were injected into the assay micro-chambers: oligomycin (Olig.), carbonyl cyanide-4-(trifluoromethoxy)phenylhydrazone (FCCP), rotenone plus antimycin A (R+A), for the OCR assay; glucose (Glc), oligomycin, 2-deoxy glucose (2DG), for the ECAR assay. Each data point is mean ± SD of four technical replicates. (**B**,**D**) are metabolic parameters inferred from the OCR and ECAR assays, respectively. The bars are mean ± SEM of three independent experiments carried out in quadruplicate under each condition and refer to the R+A-sensitive OCR and 2DG-sensitive ECAR normalized to the protein amount. In (**B**): Basal, resting OCR; oligo, OCR in the presence of oligomycin; FCCP, OCR under uncoupled condition; in the inset, the ATP-linked OCR (OCR_ATP_) and the respiratory spare capacity (i.e., uncoupled OCR – basal OCR) is also shown. In (**D**): Glycolysis, ECAR after addition of Glc; Glycolytic capacity, ECAR after addition of oligomycin; Glycolytic reserve, difference between glycolytic reserve and glycolysis. *, *P* < 0.05 and **, *P* < 0.01 vs. WT MEF. (**E**) Cellular ATP steady-state content. ATP was measured as described in the materials and methods in the absence and after 10 min following addition of 1 µM oligomycin; the oligomycin-sensitive and -insensitive ATP content are shown as stacked bars and are the means ± SEM of three biological replicates; *, *P* < 0.05 and **, *P* < 0.01. (**F**) Rates of ATP production. MEFs were assayed by Seahorse technology utilizing a specific protocol as described in the materials and methods. MitoATP is the mitochondrial OxPhos-linked production of ATP and it was estimated from the OCR corrected for the P/O ratio that was 2.25. GlycoATP is the glycolytic production of ATP and it was estimated from the ECAR following i) conversion of the pH changes in the absolute amount of H^+^ released (using a calculated buffering power of 1.79 mM H^+^/pH), and ii) correction from the CO_2_ release; see the materials and methods and reference therein for details. MitoATP and GlycoATP are shown as stacked bars and are the means ± SEM of three biological replicates; *, *P* < 0.05 and **, *P* < 0.01.

As a first line of investigation, we sought to verify if the *KMT2D* loss of function impacted the bioenergetic cell metabolism. To this aim, we used the Seahorse methodology [21] to assess the two major metabolic fluxes in intact cells: i) Mitochondrial oxidative phosphorylation (OxPhos) activity, measured as the oxygen consumption rate (OCR); and ii) glycolysis, measured as the extracellular acidification rate (ECAR) largely contributed by lactate release. Figure 1 shows the results attained from comparing the OCR and ECAR activities between wild-type (WT) MEF and *Kmt2d* - knockout (KO) MEF. The values were corrected for residual activity in the presence of the mitochondrial respiratory chain inhibitors rotenone plus antymicin A for OCR (Figure 1A) and in the presence of the glycolysis inhibitor 2-deoxyglucose for ECAR (Figure 1C). The OCR under basal conditions was significantly reduced in *Kmt2d*-KO MEF (≈70% of WT-MEF). Following addition of the H^+^-ATP synthase inhibitor oligomycin, the OCR was depressed to a similar extent in both WT-MEF and *Kmt2d*-KO MEF, indicating no variation in the membrane proton leak (Figure 1B). Addition of the protonophore uncoupler FCCP stimulated the OCR to its maximal activity in both wt-MEF and *Kmt2d*-KO MEF but to a significantly lower extent in *Kmt2d*-KO MEF (≈55% of WT-MEF) (Figure 1B). Consequently, in *Kmt2d*-KO MEF, both the OCR linked to ATP synthesis (i.e., the difference between basal OCR and OCR *plus*-oligomycin), and the spare respiratory capacity (i.e., the difference between OCR *plus*-FCCP and basal OCR) were significantly reduced (≈85% and ≈39% of wt-MEF, respectively). As compared with WT-MEF, ECAR was slightly reduced in *Kmt2d*-KO MEF under the basal condition and more significantly reduced under conditions maximally stimulating glycolysis, achieved by oligomycin treatment (i.e., glycolytic capacity) (Figure 1D). As a result, the ECAR spare capacity (i.e., glycolytic reserve) was lower in *Kmt2d*-KO MEF. Altogether, these results suggest a reduced mitochondrial OxPhos capacity in a *Kmt2d*-null background that was not apparently compensated by glycolysis, therefore pointing to a general cell bioenergetic deficiency. This conclusion was also supported by direct measurement of the cellular steady-state ATP content, whereby we found a significant 35% reduction of ATP in *Kmt2d*-KO MEF as compared with WT (Figure 1E). The ATP content was sensitive to a short-term exposure of the cells to the F_o_F_1_-ATP synthase inhibitor oligomycin, providing a measure of the OxPhos contribution and by the difference to that of glycolysis. It can be noted that the ATP contributed by the mitochondrial OxPhos system was largely inhibited in *Kmt2d*-KO MEF. Conversely, a significant increase of the oligomycin-insensitive ATP content, largely due to glycolysis and other substrate-level phosphorylations, was observed in *Kmt2d*-KO MEF. Furthermore, we measured in real time the rate of ATP production by utilizing a recently developed and validated Seahorse technology-based protocol. By comparing the rates of O_2_ consumption and of extracellular H^+^ release, simultaneously measured on the same sample, it is possible to determine the rate of ATP produced by both the OxPhos (mitoATP) and glycolysis (glycoATP) [18]. The results of this analysis, shown in Figure 1E, are in good agreement with the ATP content. Indeed, the rate of ATP produced by the mitochondrial OxPhos was significantly reduced in *Kmt2D*-KO MEF whereas that produced by glycolysis was increased. It must be highlighted that glycoATP was calculated from the actual release of H^+^ corrected from the contribution of the CO_2_ production and is therefore more reliably linked to the lactate release. We then carried out the same analysis on skin-derived fibroblast cell lines from five Kabuki patients and observed a significant reduction of OCR both under basal and maximal FCCP-stimulated respiration compared to fibroblasts from a healthy subject; additionally, the ATP-linked respiration was inhibited. Basal ECAR was depressed in both Kabuki patients’ fibroblasts, whereas the ECAR capacity was decreased in one of them (Figure S1B). 

**Figure 2:**
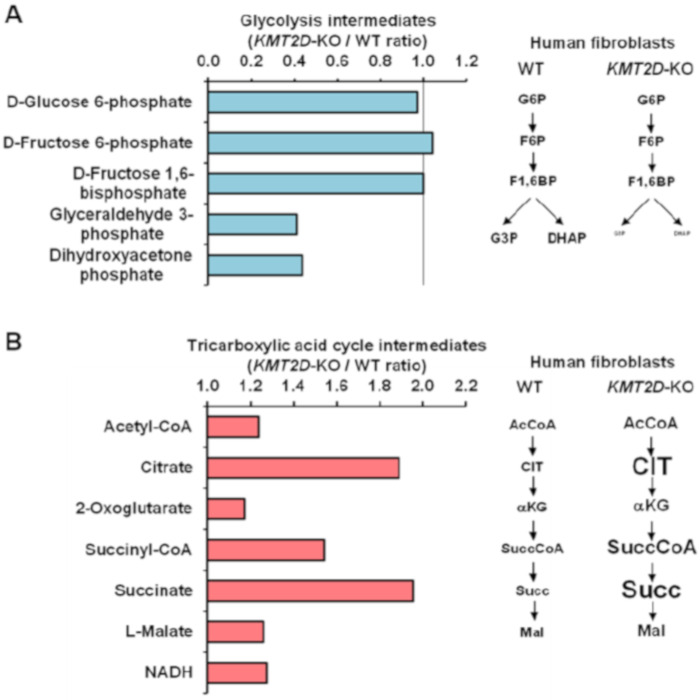
Mass spectrometry analysis of metabolites in wild type (WT) and *KMT2D*-KO fibroblasts. The histograms in (**A**,**B**) show the relative amounts of intermediate metabolites of glycolysis and the tricarboxylic acid cycle, respectively, in *KMT2D*-KO fibroblasts as compared with isogenic WT cells. The bars are averages of two biological replicates yielding similar results. The pathways on the right represent the fold change of the indicated metabolites in *KMT2D*-KO cells as compared with those in WT cells.

CRISPR-CAS9 was used to generate a knockout *KMT2D* human skin-derived fibroblast cell line (*KMT2D-KO*). The metabolite content of the generated fibroblasts clones was investigated by mass spectrometry, and the detected metabolites were clustered according to the metabolic pathways where they are involved. Figure 2 shows the relative levels of intermediates of the glycolysis and tricarboxylic acid (TCA) cycle in *KMT2D* knockdown fibroblasts when compared with the wild-type isogenic counterpart (Figure 2A,B, respectively). The results attained are consistent with the metabolic flux analysis shown in Figure 1. Indeed, glycolysis appears to be dampened in *KMT2D*-KO cells at the level of the conversion of D-fructose 1,6-bisphosphate to the trioses phosphates glyceraldehyde-3-phosphate and dihydroxyacetone phosphate, with the levels of both reduced as compared to WT cells. Notably, the detectable intermediates of the TCA cycle were all increased in *KMT2D*-KO, particularly citrate and succinate. This is consistent with the observed reduction of the mitochondrial respiratory chain and consequent re-oxidation of NADH and FADH_2_, which are necessary in the oxido-reduction steps of the TCA cycle. 

**Figure 3:**
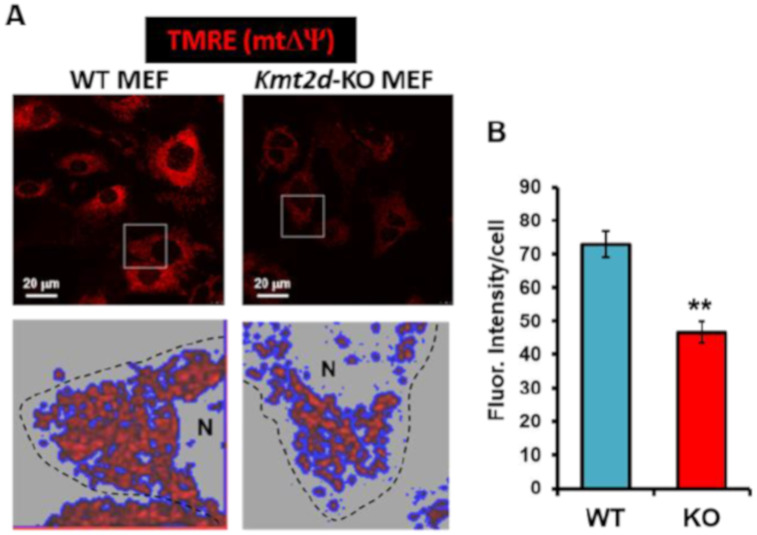
Imaging of the mitochondrial membrane potential in WT and *Kmt2d*-KO MEF. Cells pre-incubated with TMRE were analyzed by confocal microscopy. (**A**) Representative images with false-color rendering of digitalized magnifications; N, nucleus. (**B**) Quantitative analysis of the TMRE fluorescence intensity/cell expressed in arbitrary units; the bars are means ± SEM of three independent experiments; **, *P* < 0.005. For each condition, at least 10 different optical fields were randomly selected containing 10–15 cells each. See the materials and methods for further details.

The defective respiratory activity was further verified by confocal microscopy imaging of the respiration-dependent mitochondrial potential (mtΔΨ) using the specific fluorescent probe TMRE in *Kmt2d*-KO cells (Figure 3A). TMRE is a lipophilic cation that accumulates in respiring mitochondria, and this accumulation is driven by the mtΔΨ. In *Kmt2d*-KO MEF under resting conditions, the mitochondria-localized probe was significantly reduced, indicating a lower mtΔΨ as compared to its WT counterpart (Figure 3B). Notably, a closer analysis of the functional mtΔΨ-generating mitochondria revealed a more fragmented compartmentalization of the fluorescent signal in cells losing *Kmt2d* expression.

### 3.2. Loss of Kmt2d Causes Specific Downregulation of the Respiratory Chain Complexes I and IV

**Figure 4:**
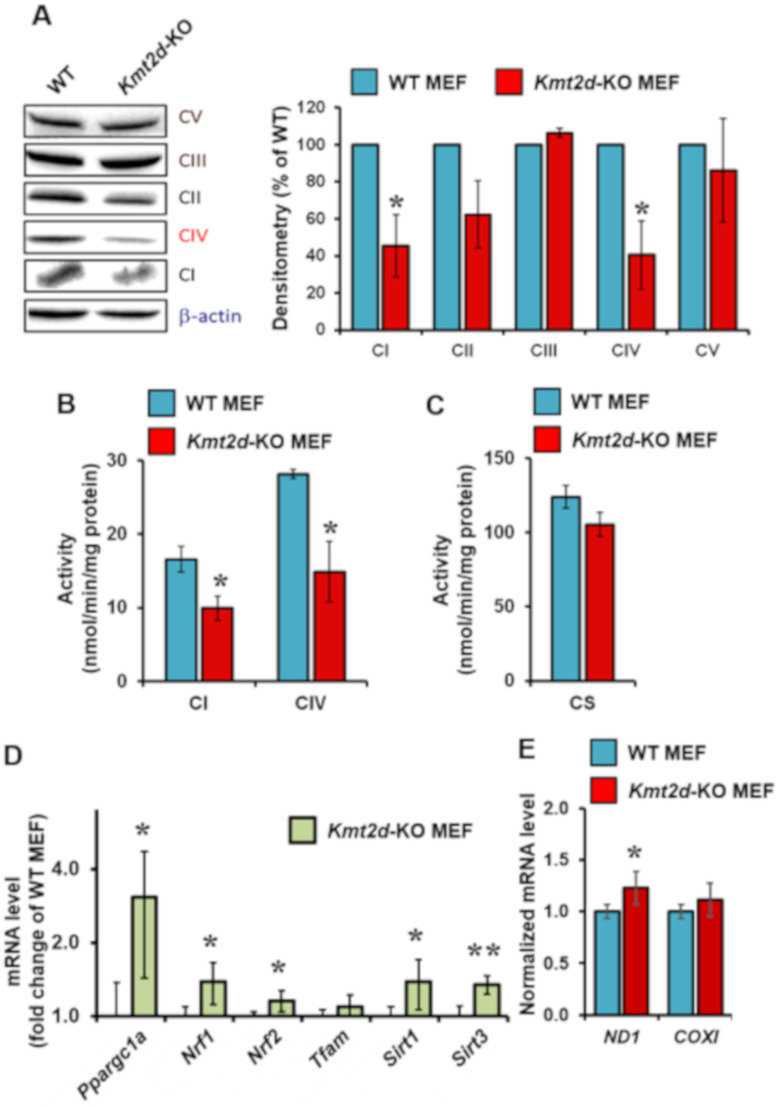
Molecular and functional analysis of the mitochondrial respiratory chain in WT and *Kmt2d*-KO MEF. (**A**) Immunoblotting of OxPhos complexes; a representative Western blotting is shown (on the left) with normalized densitometric and statistical analysis (means ± SEM of *n* = 4); *, *P* < 0.05 vs. *Kmt2d*-KO MEF. (**B**) Enzymatic activity of complex I (CI) and complex IV (CIV); (**C**) enzymatic activity of citrate synthase; in (**B**,**C**), the activities were carried out by spectrophotometry assay on cell lysate. (**D**) RT-qPCR analysis of the expression of genes coding for the indicated proteins involved in the mitochondrial biogenesis; the values are fold changes of the transcript in *Kmt2d*-MEF with respect to WT MEF (means ± SD of *n* = 3; *, *P* < 0.05; **, *P* < 0.01). (**E**) RT-qPCR analysis of the expression of mtDNA-coded genes for a subunit of complex I (ND1) and of complex IV (COXI); the values are normalized with respect to WT MEF (means ± SD of *n* = 3; *, *P* < 0.05).

Because the observed reduced activity of mitochondrial OCR under maximal stimulation could be linked to the content of the respiratory chain complexes, we analyzed the protein level of all complexes (Cs) of the OxPhos system (i.e., Cs I, II, III, IV, V) using a cocktail of antibodies recognizing a specific subunit for each complex. The results attained are illustrated in the immunoblot shown in Figure 4A and reveal a selective reduction of CI (NADH-UQ oxidoreductase/NADH dehydrogenase) and of CIV (cytochrome c oxidase) in *Kmt2d*-KO MEF as compared with WT MEF. Some reduction was also observed for CII (succinate dehydrogenase), although it did not reach any statistical significance. No difference in the protein expression of CIII (cytochrome c reductase) or CV (H^+^-F_o_F_1_ ATP synthase) was observed between the two cell samples. Determination of the specific enzymatic activity of CI and CIV by spectrophotometric assays confirmed a significant reduction of both complexes in *Kmt2d*-KO MEF compared to WT MEF (Figure 4B). Conversely, measurement of the citrate synthase activity, which is taken as an indirect marker of the mitochondrial mass, did not show significant changes (Figure 4C).

To verify if the reduced content of the respiratory chain complexes I and IV was due to defective biogenesis, we measured the expression level of the major transcription factors controlling the bi-genomic expression of the respiratory chain complexes by RT-qPCR. The expression of *Pgc1a*, which codes for the master transcription factor peroxisome proliferator-activated receptor gamma coactivator 1-alpha (PGC-1α), was three-fold higher in *Kmt2d*-KO MEF compared to WT MEF (Figure 4D). There was also a slight significant increase in the expression of the nuclear respiratory transcription factors 1/2 (*Nrf1/2*), the histone/protein deacetylases sirtuins 1/3 (*Sirt1/*3). The expression of the mitochondrial transcription factor A (*Tfam*), which is controlled by PGC-1α, was also increased, though not reaching statistical significance. Consistent with the modest effect on *Tfam* expression, which controls transcription of mtDNA genes, the expression levels of *Nd1* and *CoxI*, both coded by the mt*DNA*, were slightly increased, with statistical significance only for *Nd1* (Figure 4E). This counterintuitive result would suggest an adaptive response in *Kmt2d*-KO cells to the OxPhos dampening, which, however, did not result in a compensative outcome.

### 3.3. Loss of Kmt2d Causes Unbalance in the Reactive Oxygen Species Homeostasis

**Figure 5:**
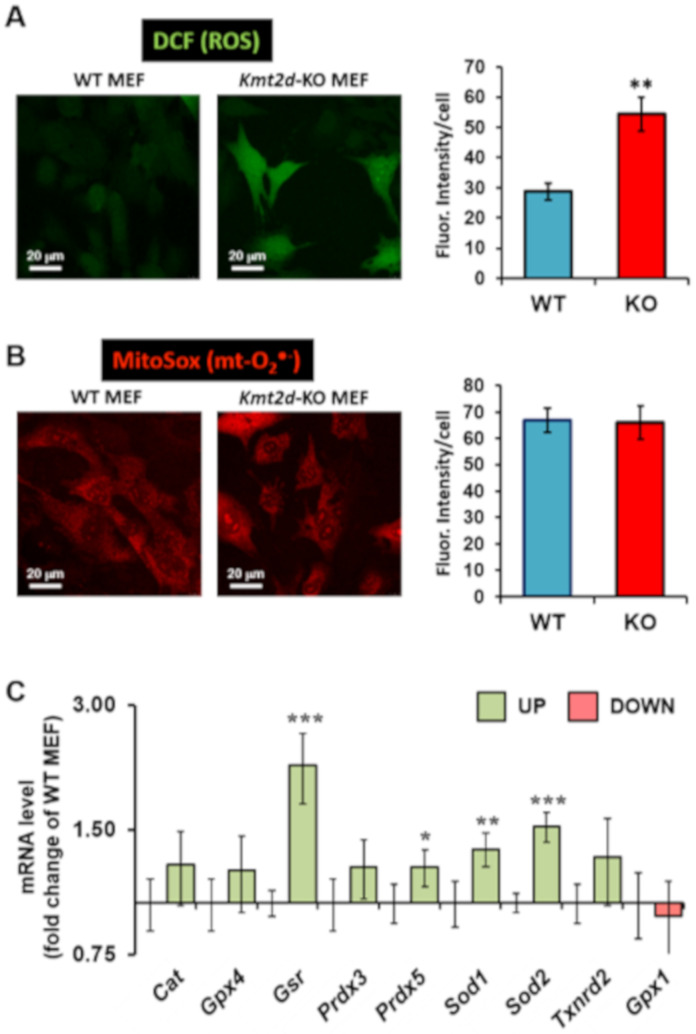
Assessment of the redox homeostasis in *Kmt2d*-KO and WT MEF. (**A**,**B**) show the confocal microscopy analysis of cells loaded with the redox-sensitive fluorescent probes DCF and MitoSox, respectively. The panels on the left are representative images, and the histograms on the right are quantification of the fluorescent signal/cell. The bars are means ± SEM of three independent experiments; **, *P* < 0.005. For each condition, at least 10 different optical fields were randomly selected containing 10–15 cells each. (**C**) RT-qPCR analysis of gene expression for the indicated proteins involved in the antioxidant defense; the values are fold changes of the transcript in *Kmt2d*-KO MEF with respect to WT MEF (means ± SD of *n* = 3; *, *P* < 0.05; **, *P* < 0.05; ***, *P* < 0.001).

Previous studies have shown that deregulation of the mitochondrial respiratory chain often leads to enhanced generation of reactive oxygen species (ROS) [22]. Therefore, we assessed the intracellular redox state by imaging *Kmt2d*-KO MEF in the presence of ROS-sensitive probes. Although the probe dichlorofluorescein (DCF) responds to a variety of reactive species, it displays some selectivity with peroxides [23]. Comparative confocal microscopy imaging and signal quantification revealed a significantly higher level of DCF-related fluorescence in *Kmt2d*-KO MEF (Figure 5A). Consistently, we observed an increase of fluorescent signal in Kabuki patients’ fibroblasts when compared to control fibroblasts (Supplementary Material Figure S2). Finally, Figure 5B shows the result attained using the probe MitoSox, which specifically detects the intramitochondrial superoxide anion species (O_2_^•−^). No difference in the MitoSox-related fluorescence signal was observed between WT MEF and *Kmt2d*-KO MEF. These findings prompted us to check the expression of genes coding for major antioxidant enzymes by using RT-qPCR (Figure 5C). Most of the selected antioxidant genes in *Kmt2d*-KO MEF were upregulated, with this increase reaching statistical significance for *Gsr* (coding for glutathione reductase), *Prdx5* (mitochondrial peroxiredoxin-5), *Sod1* (cytoplasmic Cu-Zn superoxide dismutase-1), and *Sod2* (mitochondrial Mn superoxide dismutase-2).

### 3.4. Loss of Kmt2d Changes the Glucose- and Fatty Acid-Related Metabolic Flexibility

**Figure 6:**
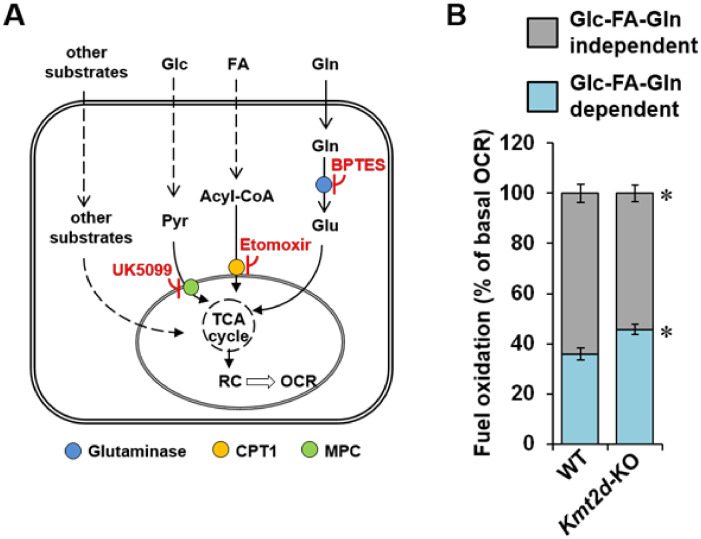
Measurement of the reliance on respiratory substrates in *Kmt2d*-KO and WT-MEF. Resting OCRs were assessed in cells by a Seahorse Analyser in the absence and in the presence of a cocktail of inhibitors (UK5099, etomoxir, and BPTS). (**A**) The targeted pathways of the inhibitors are shown; CPT1, carnitine palmitoyl transferase 1; MPC, mitochondrial pyruvate carrier; Glc, glucose; FA, long-chain fatty acids; Gln, glutamine; Glu, glutamate; Pyr, pyruvate. (**B**) Histogram showing the Glc/FA/Gln-dependent and Glc/FA/Gln-independent OCRs expressed as percentage of the rotenone + antymicin A-sensitive OCR; means ± SEM of *n* = 4 independent biological replicates in triplicate; *, *P* < 0.05.

Next, we sought to verify whether, in addition to the impaired mitochondrial respiratory chain, the loss of KMT2D function influences the reliance of the aerobic metabolism on specific oxidizable substrates. In cultured cells, the major respiratory substrates are glucose (Glc), fatty acids (FAs), and glutamine (Gln). Thus, to evaluate the link between KMT2D and these major respiratory substrates, we treated *Kmt2d*-KO MEF with a cocktail of the following drugs: UK5099, which inhibits mitochondrial pyruvate carrier (MPC); etomoxir, which inhibits carnitine palmitoyltransferase-1 (CPT-1); and BPTS, which inhibits glutaminase (Figure 6A). Figure 6B shows that this treatment resulted in a slightly higher reliance of the basal OCR on these three metabolic substrates in *Kmt2d*-KO MEF as compared with WT MEF. Other unidentified substrates, however, may contribute to the respiratory activity in both WT and *Kmt2d*-KO MEFs.

**Figure 7:**
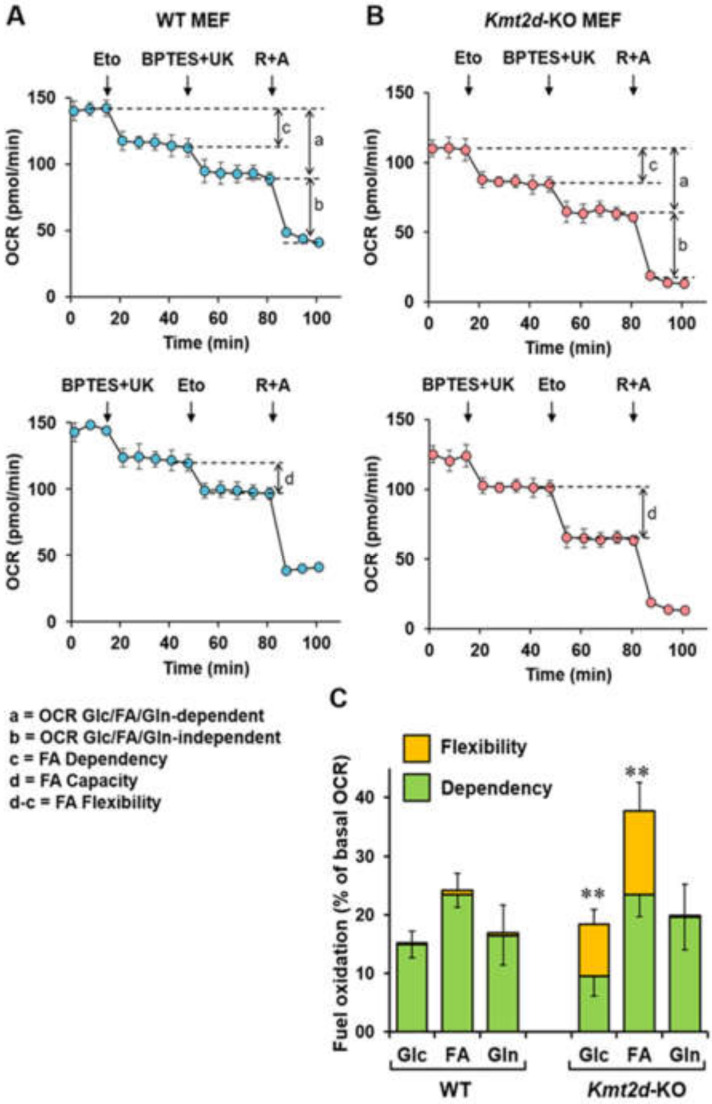
Mitofuel assay of *Kmt2d*-KO and WT MEF. Cells were assayed by the Seahorse Mito-Fuel kit as detailed in the materials and methods. (**A**) and (**B**) representative oximetric traces to assess the contribution of long-chain fatty acids (FA)s to the resting OCR. Where indicated, inhibitors of targeted pathways (see Figure 6A) were added: eto, etomoxir; BPTS + UK (5099); R+A, rotenone + antymicin A. The OCR values were utilized to extract the specific dependency, capacity, and flexibility on FA oxidation. The Glc/FA/Gln-dependent and Glc/FA/Gln-independent respiration is also shown and detailed in the legend. (**C**) Histograms showing the relative dependency and flexibility in the utilization of glucose/pyruvate (Glc), long-chain fatty acids (FAs), and glutamine (Gln) in cell respiration; the bars are mean relative values ± SEM of *n* = 4 independent biological replicates in triplicate; **, *P* < 0.01.

To better characterize the specific contributions of Glc, FA, and Gln to mitochondrial respiration, the endogenous OCR was assessed by mitochondrial fuels screening (MFS) using separate inhibitors of specific target pathways. From the MFS analysis, it is possible to estimate the dependency and the capacity of a given pathway and, by the difference between the two, its flexibility. Dependency indicates the relative amount of basal mitochondrial oxidation from a single fuel that cannot be compensated through oxidation of the other two fuels. Capacity is the relative ability of a cell to oxidize a specific fuel in the basal state when oxidation of the other two fuels is blocked. Flexibility is the difference between capacity and dependency. It indicates the relative ability of a cell in the basal state to switch or compensate mitochondrial oxidation from one fuel to another. Figure 7A,B show the representative oxymetric traces attained by MFS assay for FA oxidation in WT MEF and *Kmt2d*-KO MEF, respectively. Similar analysis of the Glc and Gln pathways revealed observable differences between *Kmt2d*-KO and WT MEF (Figure 7C). In particular, in WT MEF, the OCR dependencies from all three substrates almost coincided with their capacities but with a small but significantly higher reliance on fatty acid oxidation (FAO). In *Kmt2d*-KO MEF, the dependency on Glc was lower compared to that of FA and Gln and compared to the Glc dependency in WT MEF. However, *Kmt2d*-KO MEF exhibited larger flexibility of the Glc and FA pathways. Because of this, their Glc capacity was comparable to that of WT MEF, and the FAO capacity was even larger.

**Figure 8:**
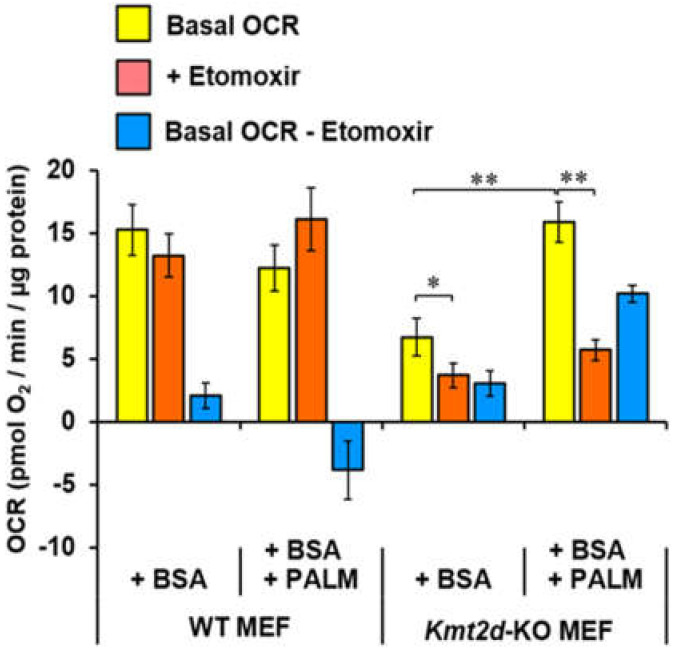
Fatty acids-related respiratory activity of *Kmt2d*-KO and WT MEF. Cells were assessed for resting OCR. Medium was supplemented with bovine serum albumin (+BSA) or with BSA + palmitate (+BSA +PALM); etomoxir was added to inhibit long-chain fatty acid oxidation. Bars are means ± SEM of *n* = 3 independent biological replicates in triplicate; *, *P* < 0.05; **, *P* < 0.01.

The higher FAO flexibility in *Kmt2d*-KO MEF was further verified by measuring the etomoxir-sensitive OCR in the presence of exogenous palmitate. Figure 8 shows that no significant difference in WT MEF OCR was observed, irrespective of the presence of FA palmitate. Conversely, the OCR in *Kmt2d*-KO MEF was lower compared to the OCR in WT MEF in the absence of FA, and it was stimulated in the presence of FA palmitate. Additionally, the OCR in *Kmt2d*-KO MEF was much more sensitive to the FAO inhibitor etomoxir. Altogether, these results suggest that, in a KMT2D-null background, a rewiring of the metabolic phenotype occurs. 

## 4. Discussion

The link between cellular metabolism and the epigenetic landscape is emerging as an important determinant of cell phenotype [1,14,24]. This notion is highlighted by increasing evidence pointing to the impact of physiological modulation or pathological alteration of given metabolic pathways on reversible modifications of DNA and histones. For instance, methylation of DNA cytosines and histone lysines, which are among the most common epigenetic modifications, all rely on S-adenosylmethionine, despite the number of different methylases involved. The regeneration of S-adenosylmethionine mainly depends on the folate cycle, which requires NADH and serine. On the other hand, demethylation by some classes of demethylating dioxygenases is tightly dependent on the availability of 2-oxoglutarate, an intermediate of the TCA cycle. Similarly, acetylation of histones requires available nuclear acetyl-CoA, which is largely generated by mitochondrial-located pathways. Conversely, histone deacetylation by sirtuins is NAD+ dependent.

Here, we investigated the impact of KMT2D on the bioenergetic cell metabolism, choosing the mitochondria as the target for our investigations. Using the Seahorse methodology, we assessed both the mitochondrial respiratory parameters and the glycolytic flux and their dependence on specific oxidizable substrates in *Kmt2d*-KO MEF. 

The results attained clearly show that the resting mitochondrial respiratory activity supported by endogenous substrates was significantly inhibited in a KMT2D-null context. Since the respiratory activity in the presence of a selective inhibitor of FoF1-ATP-synthase was scarcely affected, we can conclude that the relative portion of the OCR that translates to ATP production is negatively regulated by KMT2D. This was also confirmed by the significant reduction of the mitochondrial membrane potential, which is a measure of the respiratory chain to couple chemiosmotically their proton pumping activity to the synthesis of ATP. Notably, analysis of the glycolytic flux did not show any compensatory mechanism, which confirms the bioenergetic deficit in *KMT2D*-deficient cells. 

The observed reduced maximal OCR in *Kmt2d*-KO MEF suggested a potential reduction in the content of respiratory chain complexes. However, intriguingly, immunoblotting analysis confirmed a reduced expression of only complexes I and IV. Consistent with these findings, the enzymatic activities of complex I and IV were also reduced. In contrast, the enzymatic activity of citrate synthase was unchanged in *Kmt2d*-KO MEF. Complexes I and IV are both committed steps in the respiratory chain electron transfer, and their stalling may cause electron slippage to molecular oxygen, generating reactive oxygen species [22]. Consistently, there was a significant increase in cellular peroxides in *Kmt2d*-KO MEF. The *Kmt2d*-KO cells seemed to partially compensate for this mitochondrial dysfunction through the overexpression of antioxidant enzymes and transcription factors involved in mitochondrial biogenesis. Nevertheless, these adapting responses did not succeed in preventing the mitochondrial alterations. 

Impaired transfer of reducing equivalents to molecular oxygen is also consistent with the observed accumulation of intermediates of the TCA cycle, which depends on the re-oxidation of NADH. Moreover, a blockage of the glycolytic flux was also observed at the level of the aldolase-mediated conversion of fructose 1,6-bisphosphate to the triosephosphates. 

In addition to the defective respiratory chain, analysis of the contribution of different substrates to the OCR demonstrated a greater flexibility of *Kmt2d*-KO MEF in the utilization of pyruvate and long-chain FAs. Moreover, a relative higher capacity of FA oxidation was observed in *Kmt2d*-KO MEF, in part due to its heightened flexibility for FA oxidation. This conclusion is also supported by the observed changes in intermediates of the TCA cycle. Indeed, the accumulation of citrate in *Kmt2d*-KO MEF, wherein FA oxidation is favored, is most likely because this metabolite is normally utilized as a precursor in FA biosynthesis. Succinate accumulation may be due to enhanced production of succinyl-CoA, derived from the degradation of several amino acids and odd-chain FAs. Oxidation of succinate is also hampered by the lower availability of NAD+, which is due to lower electron transfer activity of the respiratory chain. Consequently, if oxidation of malate to oxaloacetate is hampered, this causes an accumulation of intermediates that convert back to succinate given that all reactions in this part of the TCA cycle are reversible. To note, succinate cannot be converted into succinyl-CoA because the reaction is thermodynamically unfavored, thus contributing to the accumulation of succinate. 

**Figure 9:**
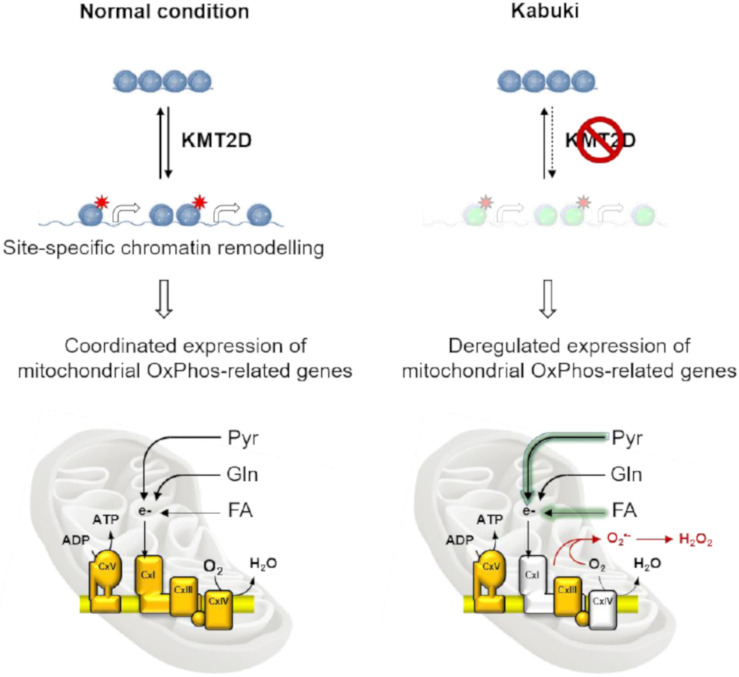
Schematic overview of the effects elicited by KMT2D on the aerobic metabolism as inferred from this study. The normal condition is shown on the left, with KMT2D-mediated chromatin remodeling controlling the coordinated expression of mitochondrial OxPhos-related genes. Alterations in the KMT2D-null context as in the Kabuki syndrome are shown on the right. Impairments include decreased expression and activity of the respiratory chain complexes I and IV with reduced oxygen consumption and the generation of reactive oxygen species. Altered flexibility in the oxidative utilization of pyruvate (Pyr) and long-chain fatty acids (FAs) is also shown as shadowed arrows; Gln, glutamine. See the discussion for further explanation.

Altogether, these results indicate that KMT2D controls the aerobic metabolism by maintaining the proper balance of enzymatic respiratory chain complexes (see the schematic drawing in Figure 9). In the last decade, convincing evidence has indicated that the respiratory chain complexes I, III, and IV are assembled in situ in supercomplexes with defined stoichiometry [25]. This would maximize the efficiency of electron transfer from NADH to dioxygen while limiting the production of reactive species [26]. Each of the three multi-subunit complexes are bi-genomic as some of their subunits are encoded by nuclear genes while others are encoded by mitochondrial DNA [27]. These two transcriptional machineries are regulated by different transcription factors. One of the best characterized transcription factors is PGC-1α, a master regulator of mitochondrial biogenesis [28,29]. This protein interacts with nuclear receptor PPAR-g, thereby allowing its interaction with multiple transcription factors [28,30]. 

Probing an already published extensive Chip-seq dataset for *Kmt2d* target genes [5], it was possible to test the involvement of them in the defective functions investigated in this study. As expected, many of the metabolic genes (involved in glycolysis and the TCA cycle) were targets of *Kmt2d* as well as genes coding for the antioxidant armory. Concerning the genes directly involved in the mitochondrial OxPhos system, with the obvious exclusion of the mitochondrial DNA genes, several nuclear genes encoding for subunits of CII, CIII, CIV, and CV were direct *Kmt2d* targets. Even if no evidence was found about CI, its decreased expression might be indirectly linked to the failure to stabilize respiratory CI-containing supercomplexes when CxIII and CIV are defective [31,32]. All this supports the deregulated oxidative metabolism reported in our study in a KMT2D-deficient context.

Consistent with the alteration of energetic metabolism in a KMT2D-null background, a number of lines of evidence are consistent with the data reported here. It has been reported that heterozygous *KMT2D*^+/−^ mice display an altered metabolic phenotype hallmarked by enhanced glucose tolerance, insulin sensitivity, and a higher serum bile acid level [12], as well as resistance to high-fat diet-induced hepatic steatosis [13]. KMT2D was consistently found to be recruited at enhancer sites by PPAR-γ, where it acted as a coactivator [33]. Thus, it was found to promote the transactivation of selected genes involved in rewiring lipid and cholesterol metabolism [13]. However, as mentioned, PPAR-γ is also involved in the homeostasis of mitochondrial function in tandem with its coactivator PGC-1α [30]. P53 is a major transcription factor found to be deregulated in a *KMT2D*-KO context [34]. In addition to its well-recognized function as a tumor suppressor due to its role in nuclear DNA repair, P53 is also involved in maintenance of the mitochondrial genome [35]. Indeed, the transcription factor A mitochondrial (*TFAM*), a gene necessary for both transcription and replication of mtDNA, has been reported to be a transcriptional target of P53 [36]. Notably, P53 also targets synthesis of cytochrome c oxidase 2 (SCO2), a factor involved in the assembly of the cytochrome c oxidase complex, thereby regulating mitochondrial respiration [37]. Using RNA-Seq analysis, it was revealed that KMT2D is, along with KMT2C, a key epigenetic regulator of the circadian clock and functions as a coactivator of the circadian transcription factors retinoid-related orphan receptor (ROR)-α and -γ [12]. Recent evidence supports a tight interplay between cell-autonomous biological clocks and metabolism, particularly with mitochondrial respiratory function [38,39]. In pancreatic carcinogenesis, transcriptional repression of KMT2D was found to be double-site CpG methylation dependent and was linked to metabolic reprogramming. In particular, the loss of H3K4me3 was associated with altered expression of the glucose transporter SLC2A3, a decreased OCR/ECAR ratio, and deregulation of FA oxidation and the lipidomic profile [40]. Loss of DPY30, a component of the ASCOM complex and facilitator of genome-wide KMT2D-mediated H3K4 methylation [41], in hematopoietic stem/progenitor cells (HSPCs) impairs the transition between pluripotent and differentiated states [42]. This was caused by impaired energy metabolism, including both glycolytic and mitochondrial pathways [43]. In a mouse model of KMT2D deficiency, early maturation of neuronal stem/progenitor cells was reported, along with strong perturbation of hypoxia-responsive metabolism pathways encompassing glycolysis, mitochondrial OxPhos, and mitochondrial transcription [44]. In the *Kmt2d*^+/bGeo^ mouse model of Kabuki, a beneficial effect of a ketogenic diet intervention was demonstrated in rescuing neurogenesis defects and hippocampal abnormalities [45]. This effect was traced back to an elevated β-hydroxybutyrate/acetoacetate ratio specifically observed in *Kmt2d*^+/bGeo^ mice. Mechanistically, it was proposed that the accumulation of β-hydroxybutyrate, an inhibitor of the histone deacetylase, counteracted the defective histone methyl transferase activity in *Kmt2d*^+/bGeo^ mice. Ketone bodies formed following a ketogenic diet are catabolic fuels serving as respiratory substrates of the mitochondrial respiratory chain. A defective respiratory chain function with stalled NADH oxidation, such as that found in our study with the *Kmt2d*-KO model, might well account for both the accumulation of upstream substrates, such as ketone bodies, and their conversion in β-hydroxybutyrate. 

## 5. Conclusions

In this study, we provided experimental evidence for a formerly unappreciated specific function of KMT2D in controlling aerobic metabolism. However, the mechanistic hints on how a KMT2D-null context affects bioenergetic metabolism remain unsolved. Although further studies are needed to understand if the reported effects are primarily linked to the *KMT2D* loss of function or mediated by secondary compensatory mechanisms, our findings may shed light on the metabolic alterations contributing to these complex disease phenotypes.

## Supplementary Materials

The following are available online at https://www.mdpi.com/2073-4409/9/7/1685/s1, Figure S1. Metabolic fluxes analysis in normal and Kabuki syndrome-affected patients’ fibroblasts, as described in the legend of Figure 1. The mutations in KMT2D carried on by each Kabuki patient are indicated in (**A**). (**A**) and (**B**) are metabolic parameters inferred from the OCR and ECAR assays, respectively. The bars are means SEM of three independent experiments carried out in triplicate under each condition. (**C**) and (**D**) are OCR- and ECAR-related parameters resulting from the averaging of the four Control and five Kabuki fibroblasts; *, *P* < 0.05 vs control fibroblasts. In the table below further features of the patients’ fibroblasts are indicated. Figure S2. Measurement of the intracellular ROS in normal and Kabuki syndrome-affected patients’ fibroblasts. DCF-loaded fibroblasts were analysed by confocal microscopy as in Figure 5A and the mean fluorescence intensity/cell of the probe was estimated as described in Materials and Methods. The bars are mean SEM of three independent experiments for each sample; *, *P* < 0.05 and ***, *P* < 0.0005 vs normal (CTRL) fibroblasts. Table S1. OFF Target prediction for gRNA used for KMT2D KO of human patient’s cell lines. (http://www.rgenome.net/cas-offinder/).
