# Supplementary material for: Loss of Function of the Gene Encoding the Histone Methyltransferase KMT2D Leads to Deregulation of Mitochondrial Respiration
Source: Cells. 2020 Jul 13;9(7):1685. doi: 10.3390/cells9071685 (PMC7407568; doi:10.3390/cells9071685)
Supplement: Supplementary file 1 [file cells-09-01685-s001.pdf]

Loss of function of the gene encoding the histone methyltransferase KMT2D leads to deregulation of mitochondrial respiration

C. Pacelli, I. Adipietro, N. Malerba, G.M. Squeo, C. Piccoli, A. Amoresano, G. Pinto, P. Pucci, J-E Lee, K. Ge,

N. Capitanio, G. Merla

Supplemental data

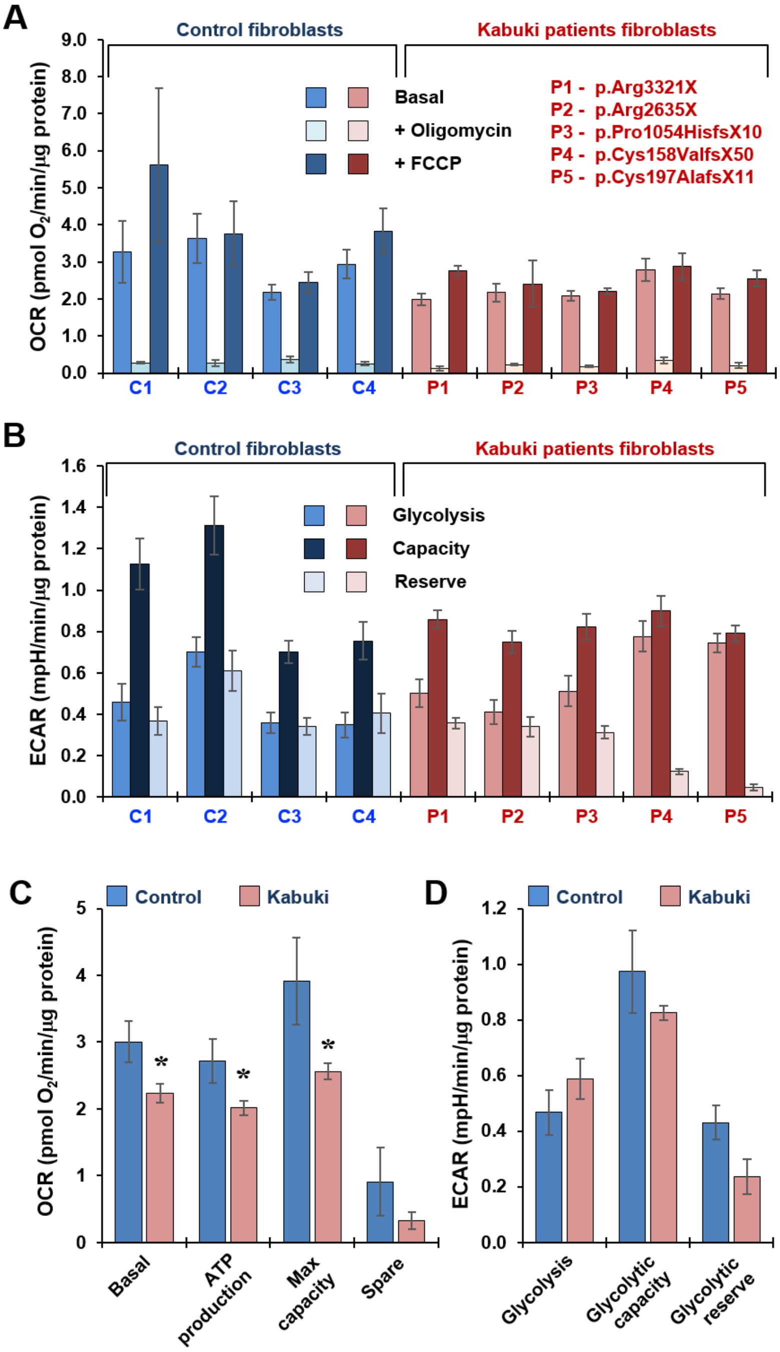

Figure S1. Metabolic fluxes analysis in normal and Kabuki syndrome-affected patients' fibroblasts, as described in the legend of Figure 1. The mutations in *KMT2D* carried on by each Kabuki patient are indicated in (**A**). (**A**) and (**B**) are metabolic parameters inferred from the OCR and ECAR assays, respectively. The bars are means ± SEM of three independent experiments carried out in triplicate under each condition. (**C**) and (**D**) are OCR- and ECAR-related parameters resulting from the averaging of the four Control and five Kabuki fibroblasts; *, $P < 0.05$ vs control fibroblasts. In the table below further features of the patients' fibroblasts are indicated.

Kabuki Patient	Sex	Age of biopsy (years)	Culture passage
#1	F	3	5
#2	F	3	5
#3	M	4	6
#4	M	5	5
#5	M	5	5

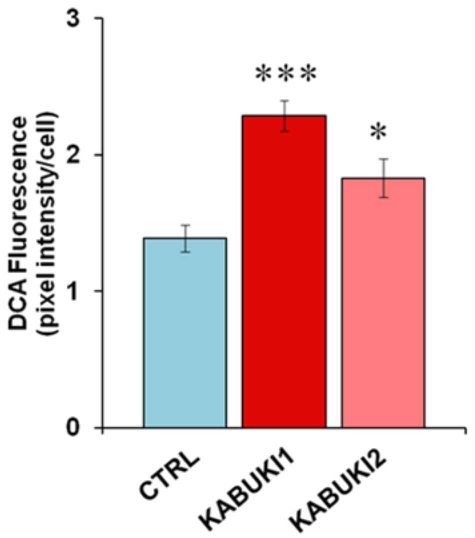

Figure S2. Measurement of the intracellular ROS in normal and Kabuki syndrome-affected patients' fibroblasts. DCF-loaded fibroblasts were analysed by confocal microscopy as in Figure 5A and the mean fluorescence intensity/cell of the probe was estimated as described in Materials and Methods. The bars are mean ± SEM of three independent experiments for each sample; *, $P < 0.05$ and ***, $P < 0.0005$ vs normal (CTRL) fibroblasts.

Table S1. OFF Target prediction for gRNA used for KMT2D KO of human patient's cell lines. (http://www.rgenome.net/cas-offinder/).

**crRNA-gRNA_A**	**DNA TARGET SEQUENCES**	**Chromosome**	**Position**	**Mismatches**
GTGCGGCGTTGTGCTCTCTGNGG	GTGCtGCGcTGTGtTCTCTGAGG	chr1	94025031	3
GTGCGGCGTTGTGCTCTCTGNGG	aTGCGGCGcTGTGtTCTCTGAGG	chr6	7840822	3
GTGCGGCGTTGTGCTCTCTGNGG	GTGCGGatTTGTGCTCTCcGGGG	chr14	100726557	3
GTGCGGCGTTGTGCTCTCTGNGG	GTGaGGCGTTGgGCTCTCTtAGG	chrX	11084607	3
GTGCGGCGTTGTGCTCTCTGNGG	GTGCtGgGTTGTtCTCTCTGTGG	chr18	48142412	3
**crRNA-gRNA_B**	DNA TARGET SEQUENCES	Chromosome	Position	Mismatches
CTCACCATTGGTGTGCTGCANGG	CTCACCATTGaTaaGCTGCAGGG	chr3	101257523	3
CTCACCATTGGTGTGCTGCANGG	CTCACCATTGGgaTGCTcCAGGG	chr1	103708618	3
CTCACCATTGGTGTGCTGCANGG	CTCACCATTGGgaTGCTcCAGGG	chr1	103643330	3
CTCACCATTGGTGTGCTGCANGG	CTCACCATTGGgaTGCTcCAGGG	chr1	103737468	3

CTCACCATTGGTGTGCTGCANGG	CTCACCATcaGTtTGCTGCATGG	chr2	87517362	3
CTCACCATTGGTGTGCTGCANGG	CTCACCATcaGTtTGCTGCATGG	chr2	111433440	3
CTCACCATTGGTGTGCTGCANGG	CTCACCcTgGGTGaGCTGCAGGG	chr17	18382352	3
CTCACCATTGGTGTGCTGCANGG	CTCAtCATTGGTcTGtTGCAAGG	chrX	38275793	3
**crRNA-gRNA_C**	DNA TARGET SEQUENCES	Chromosome	Position	Mismatches
CATGTGGGAGAGGTCTCTGTNGG	CATGTGGGAGAGtTtTCTGTTGG	chr15	57808715	2
CATGTGGGAGAGGTCTCTGTNGG	CATGTtGGAcAGGTCTCTGTTGG	chr9	117255859	2
CATGTGGGAGAGGTCTCTGTNGG	CATGTGGGAGAtGTagCTGTGGG	chr8	83559819	3
CATGTGGGAGAGGTCTCTGTNGG	aAaGTGGGAGAGaTCTCTGTGGG	chr8	130198977	3
CATGTGGGAGAGGTCTCTGTNGG	CcTGTGGGAGAGGTCcCgGTAGG	chr7	64293094	3
CATGTGGGAGAGGTCTCTGTNGG	gcTGTGGaAGAGGTCTCTGTGGG	chr5	179642176	3
CATGTGGGAGAGGTCTCTGTNGG	gcTGTGGaAGAGGTCTCTGTGGG	chr5	179652583	3
CATGTGGGAGAGGTCTCTGTNGG	CAgGTGGGAcAGGTCaCTGTGGG	chr16	29978546	3
CATGTGGGAGAGGTCTCTGTNGG	CATGTGGGgGgGcTCTCTGTGGG	chr1	1042876	3
CATGTGGGAGAGGTCTCTGTNGG	CcTGTGGGAGAGaTaTCTGTAGG	chr1	117031559	3
CATGTGGGAGAGGTCTCTGTNGG	CATGTGGGAGAGGcagCTGTGGG	chr1	155235916	3
CATGTGGGAGAGGTCTCTGTNGG	CATtTtGcAGAGGTCTCTGTCGG	chr1	208447791	3
CATGTGGGAGAGGTCTCTGTNGG	CAaGTGtGAGAGGTCTgTGTAGG	chr2	96164938	3
CATGTGGGAGAGGTCTCTGTNGG	CAgGTGGcAGAGGgCTCTGTTGG	chr19	28145548	3
CATGTGGGAGAGGTCTCTGTNGG	CAaGaGGGAGAGGTCaCTGTAGG	chr19	37345061	3
CATGTGGGAGAGGTCTCTGTNGG	CATGTGGcAGAGGTgTCTtTAGG	chr21	38603168	3
CATGTGGGAGAGGTCTCTGTNGG	CATGTGGGtGAGGTCTCaGgGGG	chr21	42186755	3
CATGTGGGAGAGGTCTCTGTNGG	CAgGTtGGAGgGGTCTCTGTGGG	chr17	74516601	3
CATGTGGGAGAGGTCTCTGTNGG	CATGTGtGAGAGGTgTtTGTGGG	chr10	12534865	3
CATGTGGGAGAGGTCTCTGTNGG	CATGTGGGAGAGGctTCTGcTGG	chr6	146676669	3
CATGTGGGAGAGGTCTCTGTNGG	CATtTGcGgGAGGTCTCTGTGGG	chr9	39364626	3
CATGTGGGAGAGGTCTCTGTNGG	CATtTGcGgGAGGTCTCTGTGGG	chr9	60923316	3
CATGTGGGAGAGGTCTCTGTNGG	CATtTGcGgGAGGTCTCTGTGGG	chr9	61198945	3
CATGTGGGAGAGGTCTCTGTNGG	CATtTGcGgGAGGTCTCTGTGGG	chr9	66983615	3
CATGTGGGAGAGGTCTCTGTNGG	CATaTGGGAGAGGTtTCTGcAGG	chr18	78098590	3
CATGTGGGAGAGGTCTCTGTNGG	CAgGaGGGAGgGGTCTCTGTAGG	chr11	67435476	3
